# Which assessment for the carriers ? The point of view of the neurologist

**DOI:** 10.1186/1750-1172-10-S1-I7

**Published:** 2015-11-02

**Authors:** Isabel Conceicão

**Affiliations:** 1 Department of Neurosciences, Centro Hospitalar Lisboa Norte - Hospital de Santa Maria and Clinical and Translational Physiology Unit, Physiology Institute, Faculty of Medicine, IMM, Lisbon, Portugal

## 

Transthyretin-related familial amyloid polyneuropathy (TTR-FAP) is a rare, autosomal-dominant, adult-onset, systemic disease caused by mutations in the transthyretin (TTR) gene that lead the TTR protein to misfold and deposit as insoluble amyloid fibrils in peripheral and autonomic nerves, the heart, and other organs [1,2].

Initial clinical symptoms may appear between the second and ninth decade of life and, without treatment, TTR-FAP leads to death on average within 10 years of symptom onset [1,2].

The disease can be difficult to recognize due to extreme phenotypic heterogeneity and nonspecific clinical symptoms.

Regular clinical surveillance for detection of first signs and symptoms of TTR-FAP in carriers should focus on clinical, neurophysiological and cardiological approaches.

Neurological involvement should be assessed by a careful clinical history looking for positive, negative sensory and motor signs and symptoms as well autonomic complaints. The use of validated clinical scales (NIS, Utah, CAD, Compass 31) and a complete small and large fibers neurophysiological evaluation should be done every year although frequency of visits should be determined on a case-by-case basis depending on clinical. Asymtomatic carriers should be evaluated once an year and symptomatic carriers under needs a closer follow-up every 6 months.

Detection of such early sign and symptoms in asymptomatic carriers establishes a diagnosis of TTR-FAP, and anti-amyloid treatment should be promptly initiated. In symptomatic patients under therapeutics a regular follow-up allows a longitudinal evaluation to reflect the maintenance of therapeutic efficacy.

## References

[B1] Ando Y Coelho T Berk JL Waddington Cruz M Goran-Ericzon B Ikeda S Guideline of transthyretin-related hereditary amyloidosis for clinicians Orphanet J Rare Dis 2013 8 31 23425518 10.1186/1750-1172-8-31 PMC3584981

[B2] Plante-Bordeneuve V Update in the diagnosis and management of transthyretin familial amyloid polyneuropathy J Neurol 2014 261 6 1227 1233 24888313 10.1007/s00415-014-7373-0

